# Exploring training-sleep characteristics and bidirectional lagged relationships in Chinese recreational runners: insights from a year-long wearable monitoring study

**DOI:** 10.3389/fphys.2026.1730135

**Published:** 2026-02-20

**Authors:** Xiaofeng Xu, Jie Lin, Kai Xu, Zhongke Gu, Xiaoming Gu, Jiaxuan Zheng, Gangrui Chen, Jiansong Dai

**Affiliations:** 1 Department of Sport and Health Sciences, Nanjing Sport Institute, Nanjing, China; 2 Sport Science Research Institute, Nanjing Sport Institute, Nanjing, China; 3 Nanjing Huipao Network Technology Co., Ltd., Nanjing, China; 4 Nanjing Paodao Network Technology Co., Ltd., Nanjing, China

**Keywords:** recreational runners, season, sleep, training, wearable devices

## Abstract

**Objective:**

This study aims to explore training and sleep characteristics among recreational runners, along with their interrelationships, to provide more scientific and personalised training guidance.

**Methods:**

Recreational runners wearing Garmin smartwatches were recruited, and continuous data on training and sleep were collected via the device Application Programming Interface from June 2024 to June 2025. Training data included pace, distance, duration, and heart rate, while sleep data included stage durations and nocturnal heart rate variability Linear mixed models were applied to examine differences in training and sleep characteristics across sex, performance classification, season, and day of the week, and to explore the effects of training load on subsequent sleep and the influence of sleep duration on training performance on the following day.

**Results:**

The average distance per training session was 12.10 ± 3.36 km, with an average pace of 6.02 ± 1.00 min/km. Male runners exhibited significantly faster paces and longer distances than females, and elite runners outperformed other groups across all training parameters. Significant seasonal and weekly variations were observed. Specifically, pace was slowest and distance shortest in summer, while training volume, intensity, and heart rate were higher on rest days than on workdays. The average nightly sleep duration was 6.61 ± 0.69 h, indicating general insufficiency. Female runners had significantly longer deep sleep than males, and elite runners showed a more pronounced early sleep-wake rhythm. A bidirectional relationship was identified, with the finding that high training loads led to reductions in deep and REM sleep, increases in light sleep and wake duration, and lower HRV, collectively indicating impaired sleep quality and autonomic recovery despite longer total sleep duration. Conversely, insufficient sleep resulted in slower pace and decreased training efficiency on the following day, though runners tended to compensate by extending running duration.

**Conclusion:**

The training and sleep characteristics of recreational runners were significantly influenced by sex, performance classification, season, and weekday-weekend rhythm. A bidirectional association between training and sleep was observed, with high training loads and insufficient sleep posing potential risks to performance and recovery. It is recommended that recreational runners monitor both training and sleep, take individual differences and temporal rhythms into account when scheduling training, and avoid excessive training loads to achieve a balance between training and recovery.

## 1 Introduction

Research indicates that regular physical activity plays a positive role in preventing chronic diseases while effectively reducing mortality rates elevated by prolonged sedentary behaviour (Andersen et al., 2016; Das and Horton, 2016). In recent years, running has gained sustained popularity as a widely embraced form of physical activity (Ding et al., 2016). Research indicates that regular participation in running activities exerts positive effects on body weight, body fat, blood pressure, blood glucose levels, insulin sensitivity, lipid profiles, and musculoskeletal health (Laye et al., 2015; Nikolaidis et al., 2021). Statistics from relevant Chinese websites indicate a substantial running enthusiast population in China, numbering 163 million, with 7.049 million participants in road running events. Regarding gender composition among participants, males remain the dominant force in road running events, accounting for 64.6%; however, the proportion of female participants is showing a sustained upward trend.

Under modern athletic training paradigms, training among recreational runners has shifted from simple experiential methods to more systematic and scientific approaches. Personalized training programs are emphasized, taking into account individual differences in physical condition, athletic ability, and training goals. These programs include balanced arrangements of training intensity, volume, and cycles. Previous studies have shown that training indicators such as frequency, volume, and running pace are important predictors of marathon performance (Doherty et al., 2020). Studies suggest elite runners exhibit higher training frequency and longer distances per session (Gordon et al., 2017). The appropriate integration of training and recovery periods induces physiological adaptations within the body, thereby enhancing athletic performance (Bestwick-Stevenson et al., 2022; Charest and Grandner, 2022). To promote recovery and improve athletic performance, professional athletes employ various methods and tools, such as nutritional supplements and performance enhancers, compression garments, massage, or cryotherapy (Barnett, 2006). For recreational runners, stretching remains a common recovery strategy. However, many overlook the critical importance of sleep. Research indicates sleep is the most From a public health perspective, regular exercise is considered beneficial for improving sleep quality. It has been reported that exercise can enhance sleep, particularly in middle-aged and older adults, as well as in individuals with chronic insomnia or depression (Zhao et al., 2023). Nevertheless, sleep patterns among many marathon runners remain concerning (Campos et al., 2022). Recreational runners are often constrained by work and daily responsibilities, leading them to train in the early morning or late evening, which may reduce total sleep duration (Feng et al., 2023). In addition, high-intensity or excessive training has been found to disturb sleep (Hausswirth et al., 2014). Sleep quality is known to promote physical recovery, reduce training-related fatigue, and prepare the body for upcoming sessions (Halson, 2016). In contrast, insufficient or poor-quality sleep may impair glycogen resynthesis, delay muscle repair, cause fatigue accumulation, increase the risk of injury, and negatively affect psychological state and training motivation (Hong et al., 2018; Sen and Tai, 2023).

Currently, some recreational recreational runners opt to emulate professional athletes’ training methods, yet this may well be the cause of frequent health issues among certain runners. Presently, systematic investigations into the training patterns and health characteristics of recreational runners remain relatively scarce. Consequently, it is imperative to conduct further in-depth exploration into the training characteristics and sleep patterns of recreational runners, along with the intrinsic relationship between these two factors. This will facilitate the development of more scientifically grounded training programmes for runners, thereby enhancing the athletic performance of recreational runners. Recreational runners often have limited training time and must balance their exercise with daily responsibilities. In addition, most do not possess the genetic, physical, physiological, or psychological advantages of professional endurance athletes. Under these circumstances, the widespread use of wearable devices provides important support for evidence-based training. Surveys have shown that more than 90% of regular runners use GPS-enabled wearables to monitor training load and improve performance (Janssen et al., 2017). Compared with traditional laboratory studies, wearable technology enables the continuous collection of multidimensional data, including both training and sleep indicators, at multiple time points with higher ecological validity, thereby providing a more accurate reflection of real-world conditions. This long-term data collection approach makes it possible to capture seasonal variations and long-term trends in the interaction between training and sleep, as well as to identify patterns related to overtraining and reduced sleep quality. In contrast to earlier studies, which were mainly short-term, cross-sectional, or based on self-reported data, continuous objective monitoring reduces recall bias and allows a more precise assessment of the actual effects of different training strategies on sleep and recovery.

In the present study, we conducted a 1-year longitudinal follow-up of recreational runners using Garmin wearable devices. Through systematic analysis of long-term data on training parameters, sleep structure, and recovery status, this study not only clarified the overall correlation trends between training and sleep at the population level but also delved into tracking the dynamics of daily training volume, sleep duration, and heart rate variability (HRV) of individual runners via case study analysis, accurately identifying training load accumulation, fluctuations in recovery indicators, and their time-lagged effects. This research design fully demonstrates the application potential of wearable data in constructing dynamic monitoring and personalized early warning systems for recreational runners, providing solid empirical support and clear practical pathways for formulating scientifically sustainable and personalized training programs for recreational runners.

## 2 Methods

### 2.1 Participants

This study was conducted in collaboration with Chinese running training platform, and participants were also recruited through online channels. The inclusion criteria for recreational runners were as follows: (1) age 18 years or older; (2) voluntary provision of personal and training information relevant to the study; (3) use of a Garmin smartwatch; and (4) consent to allow the research team to access activity data through the Garmin Health Application Programming Interface (API). Ethical approval was obtained from the Ethics Committee of Nanjing Sport University (RT-2024–20), and all participants provided written informed consent before data collection.

After screening and recruitment, a total of 224 runners successfully completed 1 year of continuous monitoring. The general characteristics of the participants are presented in Table 1. Based on the 2025 Chinese Athletics Association marathon performance grading standards, runners were classified into three performance classifications: elite, advanced, and intermediate or below. To account for physiological differences, participants were also divided into eight age groups: under 34, 35–39, 40–44, 45–49, 50–54, 55–59, 60–64, and 65 years and above (Details are described in Supplementary Appendix 1). The characteristics of the participants are shown in Table 1.

**TABLE 1:** Basic information for participants.

Characteristics	Overall	Female	Male	Elite	Advanced	Intermediate or below
n	224	42	182	78	64	82
Age (years)	39.55 ± 9.52	41.43 ± 8.75	39.12 ± 9.67	43.26 ± 8.77	39.19 ± 8.92	36.24 ± 9.51
Height (cm)	170.83 ± 6.54	163.06 ± 5.21	172.64 ± 5.40	170.35 ± 5.82	170.59 ± 7.13	171.49 ± 6.75
Body weight (kg)	65.08 ± 9.33	54.95 ± 6.47	67.45 ± 8.26	62.75 ± 6.88	64.64 ± 9.91	67.71 ± 10.32
BMI (kg/m^2^)	22.22 ± 2.20	20.64 ± 1.98	22.58 ± 2.09	21.57 ± 1.52	22.11 ± 2.19	22.93 ± 2.57

### 2.2 Data collection

Training and sleep data from participants were collected between June 2024 and June 2025. To ensure efficient data acquisition, a dedicated API interface was developed to directly access participant data. The Garmin watches used by participants were connected to mobile phones through the Garmin Connect or Garmin Connect Mobile applications. For data processing and storage, runner data were downloaded from the server backend, and individual folders were created for each participant to securely store the corresponding files. Data security was maintained through regular backups. In addition, key participant information was systematically recorded in Excel spreadsheets for subsequent data management and analysis. Due to the long monitoring period, data gaps were unavoidable. Missing data were mainly caused by participants not wearing the watches during sleep, temporary device malfunctions, or the absence of a training session on a given day. Only valid training and sleep records that were successfully captured by the devices were included in the analyses. For analyses of training and sleep characteristics, all available monitored data were used independently. When examining the relationship between training and subsequent sleep, or sleep and next-day training performance, only days with matched training-sleep data were retained, and unmatched records were excluded. No data imputation was performed. In total, 38,657 days of training data and 60,465 days of sleep data were included in the analyses. Among these, 30,655 matched days were available for analyses examining the effects of sleep on next-day training, and 30,565 matched days were used to examine the effects of training on subsequent sleep.

The training data included pace, distance, running duration, step count, cadence, average heart rate, percentage of average heart rate, maximum heart rate, and percentage of maximum heart rate. Sleep data included average and maximum nocturnal heart rate variability (HRV), sleep onset time, wake-up time, deep sleep duration, light sleep duration, awake duration, REM duration, and total sleep duration.

Training data were collected using accelerometer compensation technology during running sessions. Simultaneously, optical heart rate sensors based on photoplethysmography (PPG) enabled real-time monitoring of heart rate. During nighttime, PPG technology was employed to continuously record pulse wave signals at a sampling rate of once every 5 minutes, capturing millisecond-level variations in heartbeat intervals. The RR interval data collected overnight were analyzed using the root mean square of successive differences (RMSSD) method within the Firstbeat Analytics algorithms. These analyses generated average HRV (the mean of all valid 5-min intervals) and maximum HRV (the highest value across all 5-min intervals), providing objective indicators of recovery status. For sleep data collection, Garmin watches integrated multiple sensor inputs, including PPG and accelerometer signals. Based on Firstbeat’s neural network models, sleep stages—including light sleep, deep sleep, and REM sleep—were automatically identified, while sleep onset time, wake-up time, and the duration of each stage were accurately recorded. All collected data were dynamically calibrated using Kalman filters to ensure accuracy and reliability through sensor fusion. Third-party algorithmic models further optimized data precision and stability, and data synchronization with external applications was achieved through the Garmin Health API.

Existing studies have confirmed that Garmin devices exhibit good accuracy in measuring core running metrics such as distance, speed, and heart rate (Adams et al., 2016; Evenson and Spade, 2020). In terms of sleep monitoring, compared with the gold standard polysomnography (PSG), Garmin watches also demonstrate reliable accuracy in sleep stage classification and total sleep duration assessment (Schyvens et al., 2024). Regarding the accuracy of HRV measurement, Cassirame et al. (2017) compared HRV results detected by electroencephalography (EEG) and Garmin devices, and found no significant differences in HRV analysis under the supine position. These studies collectively validate the reliability of Garmin devices in exercise and health-related research. Additionally, a key strength of the present study lies in its long-term continuous monitoring design. Data were collected using the same device and consistent algorithms throughout the year, enabling robust intra-individual longitudinal comparisons. This design reduces reliance on absolute measurement accuracy to a certain extent, and is more conducive to conducting meaningful exploration of the temporal variation patterns and correlation characteristics between training and sleep.

### 2.3 Statistical analyses

Data were organized and analyzed using JMP 18.0 software. Linear mixed models were employed to examine training and sleep data across runners differing in gender, performance classification, season, and day of the week (Monday to Sunday). The variables “gender,” “performance classification,” “season,” and “day of the week” were treated as fixed effects, while “participant” was included as a random effect. To assess the bidirectional relationship between training and sleep, we employed a two-directional, 1-day lagged linear mixed model for separate modeling, ensuring rigorous analysis of the temporal associations between these two variables. To assess the influence of training on sleep, training intensity was categorized into three levels: light, moderate, and high volume, based on the 25th and 75th percentiles of training volume for each participant. To examine the effect of sleep on training performance, actual sleep duration was classified as follows: less than 6 h as severe sleep deprivation (Elmenhorst et al., 2008; Grandner et al., 2010; Dutil et al., 2025), 6–7 h as mild deprivation, and more than 7 h as normal sleep (Hirshkowitz et al., 2015). For the respective analyses, training condition and sleep condition were included as fixed effects, with participant as a random effect. Statistical significance was set at P < 0.05. The effect size (ηp^2^) was calculated using an F-distribution approximation according to the formula: ηp^2^ = (F × df effect)/(df error + F × df effect). Thresholds for small, medium, and large effect sizes were defined as 0.01, 0.06, and 0.14, respectively.

## 3 Results

### 3.1 Training characteristics of recreational runners

Training parameters were compared among recreational runners by gender, proficiency level, season, and day of the week. Female runners exhibited significantly slower paces and shorter distances than male runners, while no significant differences were observed for other parameters. Differences across proficiency levels were detected for pace, running duration, distance, total steps, cadence, average heart rate, and maximum heart rate. Runners with higher performance classifications demonstrated faster paces, longer single-session distances, higher cadences, and lower average and maximum heart rates during exercise.

Seasonal differences were also observed. Summer sessions were characterized by the slowest paces, shortest distances and durations, lowest total steps, and lowest cadences. In contrast, both average and maximum heart rates were notably elevated during summer compared with other seasons. Differences across days of the week were identified. Training on rest days (Saturday and Sunday) involved faster paces, longer distances and durations, and higher training intensity, as indicated by increased average heart rate, maximum heart rate, and percentage of maximum heart rate, compared with weekdays (Monday to Friday). Specific statistical results for all group comparisons, including P-values and effect sizes (η_p_^2^), are detailed in Table 2.

**TABLE 2:** Training characteristics of recreational runners.

Group	Pace (min/km)	Distance (km)	Duration (min)	Total steps (steps)	Cadence (spm)	HR_avg_ (bpm)	HR_max_ (bpm)	%HR_avg_ (%)	%HR_max_ (%)
	Mean ± SD (95%CI)	Mean ± SD (95%CI)	Mean ± SD (95%CI)	Mean ± SD (95%CI)	Mean ± SD (95%CI)	Mean ± SD (95%CI)	Mean ± SD (95%CI)	Mean ± SD (95%CI)	Mean ± SD (95%CI)
Gender									
Female	6.78 ± 1.17 (6.49,7.06)	11.06 ± 3.93 (10.18,12.19)	71.79 ± 21.41 (67.82,77.94)	11,753.88 ± 3,297.11 (11,035.75,12,756.75)	174.30 ± 10.91 (171.28,177.55)	143.72 ± 8.12 (141.04,146.31)	163.00 ± 8.47 (160.15,165.80)	76.24 ± 4.63 (74.82,77.65)	86.48 ± 5.28 (85.01,87.98)
Male	5.85 ± 0.87 (5.71,5.98)	12.34 ± 3.18 (11.90,12.85)	69.47 ± 15.67 (67.24,72.04)	11,378.58 ± 2,756.61 (10,994.09,11812,34)	174.55 ± 10.16 (173.08,176.08)	143.51 ± 8.77 (142.23,144.75)	164.42 ± 9.43 (163.05,165.75)	74.93 ± 4.64 (74.24,75.61)	85.85 ± 4.76 (85.13,86.56)
P	<0.0001	0.0373	0.2556	0.4448	0.9232	0.8896	0.3706	0.103	0.4402
F	34.277	5.058	0.645	0.586	0.2	0.0193	0.805	2.704	0.575
η_p_^2^	0.135	0.022	0.003	0.003	0.001	0.000	0.004	0.012	0.003
Level									
Elite	5.47 ± 0.53 (5.28,5.66)	14.46 ± 2.33 (13.89,15.06)	76.86 ± 12.77 (73.50,80.33)	12,521.53 ± 2,160.98 (11,940.13110.11)	177.78 ± 7.27 (175.62,179.95)	139.61 ± 11.34 (138.27,141.93)	160.96 ± 7.80 (158.95,162.95)	74.11 ± 6.70 (72.40,75.47)	85.12 ± 6.75 (84.43,86.61)
Advanced	5.89 ± 0.59 (6.68,6.10)	12.36 ± 2.63 (11.73,13.02)	71.27 ± 16.01 (67.53,75.11)	11,831.89 ± 2,659.99 (11,189.41,12,485.86)	176.11 ± 8.08 (173.73,178.52)	143.78 ± 11.94 (142.15,146.21)	165.46 ± 9.75 (163.23,167.65)	75.01 ± 6.83 (74.06,76.35)	85.98 ± 7.90 (85.07,87.47)
Intermediate or below	6.66 ± 1.21 (6.47,6.84)	9.66 ± 3.02 (9.17,10.33)	62.23 ± 17.92 (59.37,66.20)	10,129.82 ± 3,100.71 (9,630.30,10,792.69)	170.13 ± 12.58 (168.08,172.33)	146.21 ± 12.80 (144.51,148.10)	165.94 ± 13.86 (164.20,168.13)	75.80 ± 6.22 (74.84,76.90)	86.01 ± 6.35 (85.08,87.25)
P	<0.0001	<0.0001	<0.0001	<0.0001	<0.0001	<0.0001	0.0019	0.1547	0.5969
F	39.536	64.366	17.614	16.867	13.519	11.595	7.681	1.878	0.517
η_p_^2^	0.264	0.368	0.137	0.132	0.109	0.095	0.065	0.017	0.005
Season									
Spring	6.00 ± 1.55 (5.94,6.20)	11.67 ± 6.01 (11.33,12.23)	67.37 ± 38.04 (64.47,71.10)	11,103.18 ± 5,911.09 (10,879.83,11,653.30)	174.85 ± 17.02 (172.77,175.51)	142.48 ± 13.08 (141.61,143.92)	163.00 ± 14.45 (162.19,164.68)	74.65 ± 6.78 (74.14,75.39)	85.40 ± 7.50 (84.94,86.25)
Summer	6.11 ± 1.52 (6.02,6.28)	11.35 ± 5.30 (10.89,11.79)	66.97 ± 35.20 (65.14,69.73)	10,687.76 ± 5,116.24 (10,363.72,11,132.24)	172.94 ± 15.73 (171.10,173.83)	163.74 ± 13.22 (142.45,144.75)	164.35 ± 13.91 (163.86,166.33)	74.89 ± 6.55 (74.58,75.83)	86.11 ± 7.35 (85.81,87.12)
Autumn	5.83 ± 1.49 (6.02,6.28)	12.82 ± 6.91 (12.27,13.17)	72.37 ± 46.95 (70.04,74.64)	11,907.92 ± 6,852.92 (11,466.15,12,236.09)	176.28 ± 14.46 (174.15,176.88)	143.69 ± 11.72 (143.06,145.37)	163.74 ± 13.22 (163.14,165.41)	75.30 ± 6.40 (74.92,76.17)	85.81 ± 7.17 (85.45,86.76)
Winter	5.85 ± 1.33 (5.80,6.06)	12.97 ± 6.60 (12.46,13.37)	72.47 ± 37.69 (70.48,75.14)	12,176.75 ± 6,110.12 (11,856.10,12,633.40)	177.07 ± 15.52 (175.08,177.82)	142.67 ± 12.26 (142.31.144.62)	162.71 ± 13.75 (162.19,164.67)	74.78 ± 6.78 (74.51,75.76)	85.30 ± 7.57 (84.94,86.25)
P	<0.0001	<0.0001	<0.0001	<0.0001	<0.0001	<0.0001	<0.0001	<0.0001	<0.0001
F	68.99	111.271	30.292	86.78	133.804	26.573	34.033	28.202	34.224
η_p_^2^	0.005	0.009	0.002	0.007	0.010	0.002	0.003	0.002	0.003
Day									
Monday	5.94 ± 1.46 (5.94,6.21)	10.39 ± 4.86 (9.60,10.55)	59.30 ± 30.06 (56.48,61.50)	9,929.58 ± 4,674.16 (9,440.33,10,258.05)	175.69 ± 14.92 (173.36,176.19)	141.93 ± 13.19 (140.71,143.09)	161.55 ± 15.06 (159.99,162.57)	74.11 ± 6.98 (73.67,74.95)	84.35 ± 7.93 (83.79,85.14)
Tuesday	5.89 ± 1.28 (5.84,6.11)	10.66 ± 3.97 (9.97,10.88)	60.15 ± 20.97 (57.54,62.24)	9,964.72 ± 3,551.75 (9,461.09,10,238.87)	175.87 ± 13.93 (173.73,176.51)	142.60 ± 11.50 (141.89,144.22)	162.95 ± 13.19 (162.21,164.73)	74.81 ± 6.33 (74.29,75.55)	85.49 ± 7.25 (84.97,86.29)
Wednesday	5.81 ± 1.25 (5.75,6.02)	11.08 ± 5.00 (10.34,11.25)	62.08 ± 28.28 (59.01,63.73)	9,828.85 ± 4,489.42 (9,169.65,9949.27)	175.13 ± 14.43 (172.76,175.54)	143.54 ± 11.94 (142.85,145.18)	165.33 ± 13.91 (164.76,167.28)	75.18 ± 6.48 (74.81,76.07)	86.60 ± 7.46 (86.29,87.62)
Thursday	5.89 ± 1.34 (5.84,6.11)	10.68 ± 4.24 (9.88,10.80)	60.48 ± 23.66 (57.20,61.96)	9,913.68 ± 3,950.84 (9,289.23,10,074.63)	175.71 ± 14.33 (173.32,176.11)	142.65 ± 11.77 (141.85,144.19)	163.20 ± 13.71 (162.50,165.03)	75.75 ± 6.45 (74.28,75.55)	85.52 ± 7.42 (85.12,86.45)
Friday	6.00 ± 1.63 (5.92,6.19)	10.65 ± 4.84 (9.87,10.80)	61.80 ± 35.17 (58.34,63.33)	10,084.38 ± 4,913.27 (9,512.31,10,311.34)	174.47 ± 18.37 (172.72,175.53)	141.35 ± 12.52 (141.12,143.47)	161.57 ± 14.07 (161.29,163.84)	74.09 ± 7.64 (73.89,75.16)	84.70 ± 7.51 (84.48,85.82)
Saturday	6.14 ± 1.77 (6.05,6.32)	14.07 ± 7.25 (13.59,14.51)	83.80 ± 55.40 (81.64,86.40)	13,597.49 ± 7,389.41 (13,262.78,14,047.29)	174.26 ± 17.98 (172.36,175.15)	142.61 ± 12.33 (142.12,144.46)	163.16 ± 13.49 (162.69,165.22)	74.84 ± 7.14 (74.40,75.67)	85.62 ± 7.02 (85.18,86.51)
Sunday	5.98 ± 1.57 (5.89,6.16)	17.20 ± 8.49 (17.02,17.93)	97.42 ± 50.80 (96.48,101.17)	16,369.19 ± 7,967.25 (16,231.35,17,007.54)	175.69 ± 15.94 (173.71,176.49)	145.54 ± 12.69 (144.85,147.18)	165.87 ± 13.15 (165.03,167.54)	76.31 ± 7.72 (75.85,77.11)	86.97 ± 6.96 (86.44,87.77)
P	<0.0001	<0.0001	<0.0001	<0.0001	<0.0001	<0.0001	<0.0001	<0.0001	<0.0001
F	26.948	1,108.872	746.794	1,114.473	7.431	79.921	88.82	81.26	89.735
η_p_^2^	0.004	0.148	0.104	0.148	0.001	0.012	0.014	0.013	0.014

### 3.2 Sleep characteristics of recreational runners

Sleep parameters were compared among recreational runners by gender, performance classification, season, and day of the week. Female runners were found to have earlier sleep onset times, longer deep sleep durations, and shorter total sleep durations compared with male runners. In contrast, male runners exhibited shorter awake durations. Significant differences were also observed across runners of varying performance classifications. Runners with higher performance classifications showed higher HRV values, earlier sleep onset times, and earlier wake-up times, indicating a more pronounced early sleep-wake rhythm.

Seasonal variations in sleep characteristics were identified. Both average and peak HRV values were highest in summer and lowest in winter. Total sleep duration was longest in winter and shortest in summer. Differences in sleep patterns were also found across days of the week, reflecting variations in sleep onset time, wake-up time, and the durations of deep sleep, light sleep, REM sleep, and awake periods between weekdays and rest days. Specific statistical results for all group comparisons, including P-values and effect sizes (η_p_^2^), are detailed in Table 3.

**TABLE 3:** Sleep characteristics of recreational runners.

Group	Average HRV	Maximum HRV	Sleep onset time (h:min)	Deep sleep (min)	Light sleep (min)	REM (min)	Awake (min)	Total sleep duration (hour)	Wake-up time (h:min)
	Mean ± SD (95%CI)	Mean ± SD (95%CI)	Mean ± SD (95%CI)	Mean ± SD (95%CI)	Mean ± SD (95%CI)	Mean ± SD (95%CI)	Mean ± SD (95%CI)	Mean ± SD (95%CI)	Mean ± SD (95%CI)
Gender									
Female	56.61 ± 17.32 (49.12,61.75)	89.81 ± 23.92 (81.64,99.52)	23:39±:1:14 (23:02,23:39)	84.00 ± 28.80 (98.40,124.20)	253.80 ± 33.60 (229.80,252.60)	55.80 ± 27.00 (45.60,63.60)	22.27 ± 23.07 (57.90,125.34)	6.55 ± 0.66 (6.55,6.95)	6:35 ± 1:27 (6:04,6:47)
Male	55.68 ± 15.83 (53.79,59.45)	91.75 ± 26.09 (85.84,93.81)	23:26±:1:26 (23:28,23:47)	111.60 ± 76.80 (77.40,90.00)	241.80 ± 47.40 (247.80,259.20)	55.80 ± 36.00 (51.60,60.00)	15.07 ± 10.13 (115.32,148.20)	6.82 ± 0.77 (6.45,6.64)	6:30±:1:30 (6:24,6:45)
P	0.7885	0.6953	0.0496	0.0002	0.0536	0.7999	0.0497	0.0224	0.254
F	0.072	0.154	3.899	14.956	3.535	0.001	3.897	5.292	1.308
η_p_^2^	0	0.001	0.018	0.066	0.016	0	0.018	0.024	0.006
Level									
Elite	58.66 ± 17.77 (54.25,63.09)	94.87 ± 25.24 (88.67,101.04)	23:15±:1:10 (23:01,23:28)	82.20 ± 33.00 (72.00,92.40)	252.00 ± 34.80 (246.00,261.00)	54.00 ± 26.40 (47.40,60.60)	20.51 ± 12.66 (16.21,24.84)	6.47 ± 0.66 (6.32,6.62)	6:10±:1:24 (5:24,6:25)
Advanced	54.93 ± 14.02 (50.04,59.69)	88.07 ± 21.63 (80.09,94.42)	23:38±:1:21 (23:21,23:50)	100.20 ± 64.20 (89.40,50.40)	242.40 ± 43.80 (233.40,252.00)	53.40 ± 31.80 (46.20,60.60)	18.81 ± 14.48 (14.19,23.46)	6.60 ± 0.77 (6.41,6.73)	6:33±:1:31 (6:15,6:49)
Intermediate or below	55.62 ± 18.44 (51.35,59.79)	87.44 ± 24.90 (81.29,93.11)	23:50±:1; 08 (23:38,00:04)	87.00 ± 29.40 (77.40,96.60)	257.40 ± 32.40 (248.40,264.60)	59.40 ± 28.80 (52.80,65.40)	22.73 ± 30.08 (18.18,26.33)	6.73 ± 0.64 (6.55,6.84)	6:50±:1:14 (6:40,7:10)
P	0.4699	0.1867	0.0016	0.1023	0.1072	0.591	0.7286	0.1769	0.0002
F	0.786	1.869	7.236	3.004	2.638	0.89	0.604	2.201	3.188
η_p_^2^	0.006	0	0.001	0	0.026	0.124	0.044	0.001	0.05
Season									
Spring	55.05 ± 17.96 (52.28,57.45)	87.03 ± 25.59 (83.76,91.01)	23:37 ± 1:16 (2:26,23:42)	510.00 ± 235.56 (68.28,94.20)	1,504.44 ± 300.36 (244.20,254.40)	358.32 ± 191.70 (54.48,61.80)	19.03 ± 17.06 (17.19,22.19)	6.58 ± 0.96 (6.50,6.68)	6:35 ± 1:26 (6:23,6:420
Summer	59.74 ± 19.18 (56.83,61.99)	95.79 ± 27.00 (91.91,99.15)	23:41 ± 1:15 (23:32,23:48)	501.12 ± 187.56 (82.80,94.80)	1,479.36 ± 288.60 (239.40,249.60)	312.66 ± 185.70 (46.80,54.60)	20.22 ± 18.3 (18.18,23,18)	6.37 ± 0.93 (6.31,6.48)	6:50 ± 1:27 (6:17,6; 36)
Autumn	58.10 ± 18.83 (55.57,60.73)	92.58 ± 26.84 (89.16,96.41)	23:30 ± 1:14 (23:20,23:46)	511.86 ± 201.06 (84.60,96.00)	1,519.38 ± 300.06 (246.60,256.80)	329.40 ± 193.32 (50.40,57.60)	20.33 ± 18.12 (18.25,23.25)	6.56 ± 0.96 (6.50,6.67)	6:28 ± 1:28 (6:18,6:37)
Winter	52.91 ± 17.26 (50.11,55.28)	82.98 ± 24.88 (79.56,86.81)	23:41 ± 1:18 (23:27,23:44)	501.42 ± 222.18 (82.80,94.20)	1,565.40 ± 308.10 (254.40,264.60)	375.30 ± 205.20 (56.40,64.20)	20.95 ± 19.49 (19.1,24.11)	6.78 ± 1.01 (6.71,6.89)	6:26 ± 1:26 (6:39,6:58)
P	<0.0001	<0.0001	<0.0001	<0.0001	<0.0001	<0.0001	<0.0001	<0.0001	<0.0001
F	1,236.739	1,102.22	70.521	11.255	161.984	316.407	16.593	277.431	270.17
η_p_^2^	0.072	0.064	0.003	0.001	0.008	0.016	0.001	0.014	0.013
Day									
Monday	56.04 ± 18.50 (53.46,58.64)	89.39 ± 26.54 (86.22,93.53)	23:35 ± 1:21 (23:26,23:43)	503.34 ± 212.58 (81.60,93.60)	1,534.68 ± 307.02 (252.00,262.20)	346.74 ± 194.76 (57.60,60.00)	20.24 ± 18.79 (18.25,23.30)	6.66 ± 0.97 (6.57,6.75)	6:36 ± 1:34 6:29,6:48 ()
Tuesday	57.60 ± 18.56 (54.94,60.11)	91.13 ± 26.55 (87.72,95.03)	23:36 ± 1:16 (23:23,23:39)	497.40 ± 200.28 (83.40,94.80)	1,550.52 ± 298.98 (245.40,255.60)	353.16 ± 195.84 (53.40,61.20)	19.48 ± 18.24 (17.58,22.63)	6.59 ± 0.95 (6.5,6.69)	6:39 ± 1:22 (6:20,6:39)
Wednesday	56.56 ± 18.54 (53.88.59.05)	89.70 ± 26.77 (86.29,93.60)	23:33 ± 1:13 (23:27,23:43)	504.42 ± 206.16 (84.00,96.00)	1,513.26 ± 298.92 (243.60,253.80)	344.52 ± 194.16 (52.20,60.00)	19.56 ± 20.42 (17.41,2.46)	6.56 ± 1.01 (6.47,6.65)	6:30 ± 1:24 (6:21,6:40)
Thursday	56.18 ± 18.46 (53.61,58.78)	88.93 ± 26.42 (85.67,92.98)	23:38 ± 1:17 (23:27,23:43)	508.92 ± 218.22 (84.00,95.40)	1,502.88 ± 297.54 (243.60,253.80)	340.26 ± 196.32 (51.00,58.80)	19.90 ± 18.11 (17.01,22.96)	6.53 ± 0.93 (6.46,6.68)	6:33 ± 1:27 (6:22,6:41)
Friday	56.33 ± 18.46 (53.82,58.99)	89.64 ± 26.70 (86.43.93.74)	23:37 ± 1:14 (23:27,23:43)	510.72 ± 205.74 (82.80,94.80)	1,501.26 ± 295.80 (244.80,270.00)	343.44 ± 195.72 (51.60,59.40)	19.81 ± 18.21 (17.92,22.97)	6.54 ± 0.96 (6.47,6.65)	6:33 ± 1:28 (6:23,6:42)
Saturday	56.41 ± 18.42 (53.80,58.97)	89.28 ± 26.55 (85.94.93.25)	23:37 ± 1:17 (23:29,23:46)	503.70 ± 217.98 (84.00,95.40)	1,507.56 ± 295.68 (244.20,254.40)	340.32 ± 197.98 (51.00,58.80)	20.60 ± 18.63 (18.74,23.80)	6.55 ± 1.04 (6.46,6.64)	6:34 ± 1:22 (6:26,6:45)
Sunday	56.06 ± 18.60 (53.37,58.55)	89.23 ± 26.39 (85.78,93.09)	23:40 ± 1:17 (23:24,23:41)	509.46 ± 222.90 (82.80,94.80)	1,508.40 ± 309.00 (249.00,319.20)	336.00 ± 195.30 (50.40,58.20)	21.42 ± 20.82 (19.27,24.31)	6.60 ± 1.04 (6.52,6.70)	6:37 ± 1:32 (6:25.6:44)
P	<0.0001	<0.0001	<0.0001	<0.0001	<0.0001	<0.0001	<0.0001	<0.0001	<0.0001
F	19.623	9.514	7.823	5.428	24.578	8.253	6.903	10.93	14.767
η_p_^2^	0.002	0.001	0.001	0.001	0.002	0.001	0.001	0.001	0.001

### 3.3 Interaction between training and sleep

Training intensity was categorized into three groups: light training, moderate training, and high-volume training, based on the 25th and 75th percentiles of each runner’s running volume. Significant variations in sleep patterns were observed across different training intensities. As training intensity increased from light to high, marked changes in sleep architecture were detected: deep sleep duration and REM sleep duration decreased, while light sleep duration and awake duration increased. Total sleep duration, however, showed a slight upward trend. Specific statistical results for all group comparisons, including P-values and effect sizes (η_p_^2^), are detailed in Table 4.

**TABLE 4:** Effects of training volume on sleep.

Variables	Light	Moderate	High	P	F	η_p_^2^
	Mean ± SD (95%CI)	Mean ± SD (95%CI)	Mean ± SD (95%CI)			
Deep sleep (min)	86.54 ± 29.47 (82.28,94.09)	87.38 ± 45.33 (81.42,93.20)	86.17 ± 47.59 (80.07,91.88)	<0.0001	10.582	0.001
Light sleep (min)	250.55 ± 37.77 (244.17,254.67)	251.80 ± 39.75 (246.53,256.88)	259.80 ± 42.56 (255.46,265.97)	<0.0001	86.503	0.006
REM (min)	54.24 ± 29.16 (50.96,58.53)	54.47 ± 28.06 (50.24,57.76)	52.77 ± 27.87 (49.59,57.16)	0.0113	4.480	0.000
Awake (min)	19.45 ± 13.14 (17.43,21.26)	19.65 ± 13.77 (17.90,21.65)	21.92 ± 15.50 (20.20,24.02)	<0.0001	32.736	0.002
Total sleep duration (h)	6.52 ± 0.69 (6.43,6.62)	6.53 ± 0.70 (6.45,6.63)	6.65 ± 0.75 (6.56,6.75)	<0.0001	31.719	0.002
Average HRV	56.49 ± 17.59 (53.72,58.96)	56.02 ± 17.22 (53.47,58.70)	54.82 ± 17.19 (52.14,57.38)	<0.0001	52.549	0.004
Maximum HRV	90.00 ± 24.56 (86.12,93.43)	89.16 ± 24.10 (85.60,92.87)	87.58 ± 23.62 (84.12,91.43)	<0.0001	19.912	0.002

Actual sleep duration was classified into three categories: less than 6 h as severe sleep deprivation, 6–7 h as mild deprivation, and more than 7 h as normal sleep. Sleep duration was found to significantly influence training performance. Runners experiencing sleep deprivation showed longer running durations, greater distances covered, and higher total step counts in subsequent training sessions compared with those who were well rested. Specific statistical results for all group comparisons, including P-values and effect sizes (η_p_^2^), are detailed in Table 5.

**TABLE 5:** Effects of different sleep durations on training.

Variables	Severe deprivation	Mild deficiency	Normal sleep	P	F	η_p_^2^
	Mean ± SD (95%CI)	Mean ± SD (95%CI)	Mean ± SD (95%CI)			
Pace(min/km)	6.15 ± 1.16 (5.95,6.23)	6.04 ± 1.07 (5.88,6.16)	6.00 ± 1.10 (5.83,6.11)	<0.0001	15.686	0.001
Distance (km)	13.24 ± 4.70 (12.78,13.73)	12.23 ± 3.75 (11.76,12.69)	11.35 ± 3.51 (10.76.11.70)	<0.0001	159.444	0.010
Duration (min)	78.09 ± 28.14 (74.84,79.67)	70.51 ± 18.98 (68.12,72.88)	64.89 ± 17.99 (61.69,66.69)	<0.0001	158.867	0.010
Total steps	12,575.45 ± 4,274.21 (12,131.63,12,940.43)	11,556.85 ± 3,209.29 (11,136.72,11,936.24)	10,736.40 ± 3,156.12 (10,178.91,10,984.73)	<0.0001	155.693	0.010
Cadence (spm)	173.45 ± 11.12 (172.40.175.27)	174.28 ± 11.51 (173.07,175.92)	174.73 ± 11.46 (173.43,176.30)	<0.0001	9.680	0.001
HR_a_ᵥg (bpm)	143.60 ± 9.48 (142.09,144.48)	143.77 ± 9.49 (142.46,144.84)	143.41 ± 9.35 (142.09,144.48)	0.0004	7.875	0.001
HR_max_ (bpm)	164.76 ± 10.18 (163.81,166.41)	164.36 ± 9.83 (163.02,165.60)	163.66 ± 9.96 (162.10,164.69)	<0.0001	30.284	0.002
%HR_a_ᵥg (%)	75.27 ± 4.50 (74.82,76.12)	75.27 ± 5.00 (74.62,75.92)	75.05 ± 5.30 (74.43,75.73)	0.0002	8.425	0.001
%HR_max_ (%)	86.36 ± 5.22 (85.83,87.20)	86.06 ± 5.11 (85.41,86.77)	85.63 ± 5.50 (84.92,86.28)	<0.0001	31.805	0.002

### 3.4 Individual-level longitudinal case analysis of training load, sleep duration, and HRV

**FIGURE 1:**
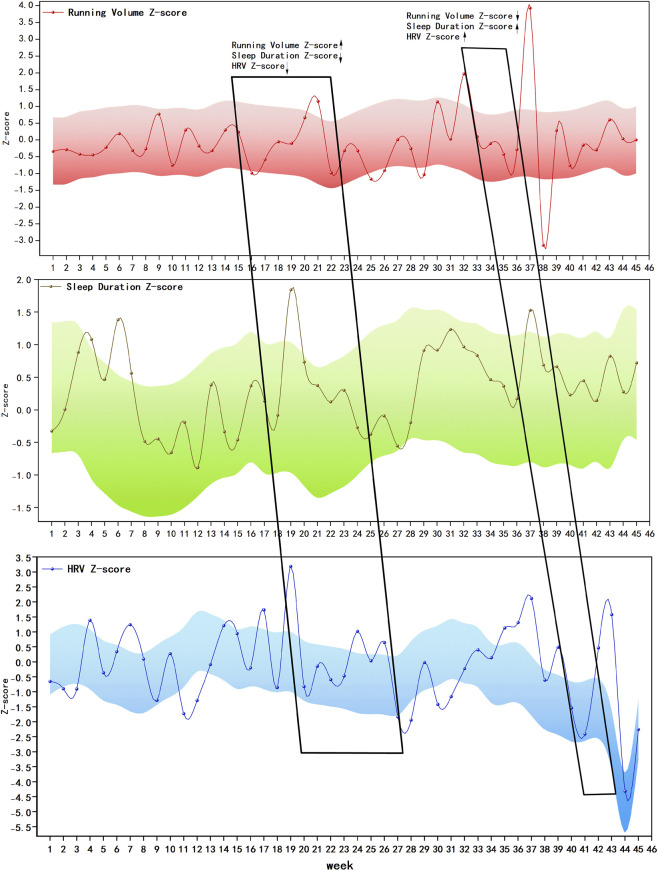
Standardized dynamic variation plot of training load, sleep duration, and nocturnal HRV in an individual runner.

To further characterize the dynamic variation features of training volume, sleep duration, and nocturnal heart rate variability (HRV) at the individual level, this study selected a 39-year-old male runner as a representative case for longitudinal analysis. Figure 1 presents the time-series changes of synchronously collected training volume, sleep duration, and HRV data from the runner over 200 valid running training days within 1 year. Given the differences in dimensions and distribution characteristics among training volume, sleep duration, and HRV, Z-score standardization was applied to these indicators to uniformly present their dynamic trends and enhance comparability. Using a 28-day rolling window as the reference, the study iteratively calculated the average training volume, average sleep duration, and average HRV of the runner over each consecutive 28-day period (e.g., Days 1–28, Days 2–29, Days 3–30, and so on) to characterize the long-term (chronic) levels of his training load and recovery status. On this basis, the Z-score of each daily observation relative to its corresponding 28-day rolling mean was further calculated to quantify the degree of deviation of short-term (acute) changes from the individual’s long-term baseline.

Furthermore, the mean value of Z-scores over 28 consecutive days plus or minus 1 standard deviation was defined as the runner’s individualized normal range, which is indicated by the shaded area in Figure 1. Considering the runner’s cumulative 200 valid records within 1 year, to avoid the visualization of overall trends being affected by high-density data points, the study further calculated the weekly average Z-scores of training volume, sleep duration, and HRV, thereby presenting their overall variation trajectories over 45 weeks. As shown in the left box of Figure 1, during a certain period, when the training volume continuously increased relative to the individual’s baseline, sleep duration and HRV exhibited a synchronous downward trend. This pattern suggests that when short-term training load increases significantly and forms a certain degree of cumulative effect, the runner may experience temporary fatigue accumulation, with physiological manifestations of shortened sleep duration and suppressed HRV. This combined pattern of increased training load accompanied by transient impairment of recovery indicators is consistent with the typical physiological response characteristics of functional overreaching. In contrast, as shown in the right box of Figure 1, when the training volume decreased significantly during a certain period, sleep duration increased accordingly, and HRV also showed an upward trend. This phenomenon indicates that after adequate recovery (usually several days to 1–2 weeks), the runner may experience supercompensation, which is characterized by improved sleep recovery and increased HRV, thereby creating favorable physiological conditions for the improvement of subsequent athletic performance.

In addition, this case study revealed a certain time-lagged effect between changes in training load and responses of recovery indicators. The significant increase in short-term training volume does not immediately lead to shortened sleep duration and obvious decline in HRV; instead, it often requires a certain period of continuous load accumulation. This result suggests that functional overreaching is more likely to be induced by persistent accumulation of training load rather than short-term, occasional fluctuations in training intensity.

## 4 Discussion

### 4.1 Training characteristics of recreational runners

Training plays a pivotal role in marathon performance. Data from 224 recreational runners were tracked over 1 year, revealing that the average training pace for these runners was 6.02 ± 1.00 min/km, with an average session duration of 69.90 ± 16.87 min, average training distance of 12.10 ± 3.36 km, total steps of 11,448.95 ± 2,861.46, and average cadence of 174.50 ± 10.28 steps per minute. Further analysis revealed statistically significant differences across multiple training metrics between recreational runners of different genders and fitness levels. Male recreational runners recorded an average pace of 5.85 ± 0.87 min/km and a single-day running distance of 12.34 ± 3.18 km, while females averaged 6.78 ± 1.17 min/km and 11.06 ± 3.93 km. These findings are consistent with previous studies, which reported that male runners generally engage in greater training volumes than female runners (Knechtle et al., 2021). This difference may reflect distinct training strategies, with female runners favouring slower paces and moderate distances to prioritise endurance development, while male runners tend to adopt higher paces and longer distances (Larumbe-Zabala et al., 2019). Gender-specific motivations may also contribute, as women often participate in long-distance running for social interaction and enjoyment rather than competitive goals (Boudreau, 2006; Crofts et al., 2012).

When runners were classified by performance classification based on marathon completion times, pace was observed to decrease as athletic proficiency declined. Concurrently, running duration, distance, step count, and cadence exhibited graded reductions. Previous studies indicate that training frequency and running speed are key determinants of marathon pace improvement (Knechtle et al., 2011; Fokkema et al., 2020). Furthermore, greater accumulated long-distance experience and higher weekly training volumes were associated with smaller declines in pace during the latter stages of a marathon and more stable pacing throughout the race (Swain et al., 2020). Accordingly, Recreational runners seeking to improve marathon finishing times should be encouraged to progressively increase running distances and training paces in their regular training regimens.

Recreational runners exhibit distinct training patterns across seasons. During summer, running pace, distance covered, and exercise duration are significantly reduced. In hot conditions, training volume may be voluntarily decreased to prevent excessive fatigue and heat-related risks, as high temperatures reduce tolerance for discomfort. In contrast, all training metrics improve in autumn and winter, with faster paces, longer distances, and increased total steps, indicating that training intensity and volume rebound under cooler conditions. Thermal adaptation to hot environments has been shown to enhance cardiac output and regulate vasodilation, facilitating efficient blood redistribution to the skin for heat dissipation while maintaining vital organ perfusion. Improved thermoregulatory capacity allows stable physiological function across a wider temperature range, reducing fatigue and performance decline due to overheating. Heat-acclimatised athletes are able to maintain thermoregulatory balance more effectively, avoiding performance reductions in hot conditions and enhancing overall athletic performance (Aoyagi et al., 1997; Racinais et al., 2015). Training in hot conditions has also been reported to improve running endurance among personnel monitored across all seasons (Dhahbi et al., 2018). Nevertheless, appropriate hydration and heart rate monitoring are essential to prevent dehydration and ensure safety during exercise (Racinais et al., 2015).

Recreational runners differ from professional athletes, as most balance work commitments from Monday to Friday and train during remaining leisure hours. Weekly training patterns reflect this schedule, with sessions on Saturday and Sunday showing significantly greater distance, total steps, and duration. During weekdays, training is often limited to commuting or brief breaks, resulting in compressed training volumes. Conversely, rest days provide more discretionary time, allowing runners to extend duration and distance to achieve desired training loads (Wasityastuti, 2021). Physiological monitoring indicates higher heart rates on rest days, suggesting increased training intensity. This variation in training between workdays and rest days has dual implications. Increased activity on rest days contributes to improvements in cardiorespiratory fitness and endurance capacity. However, excessively prolonged single sessions or high training volumes may elevate the risk of overtraining and injury. Previous studies have identified variations in training volume and load as a common cause of running-related injuries (Damsted et al., 2019; Filipas et al., 2022). To promote sustained healthy development, high-intensity interval training (HIIT) can be scheduled during weekdays using fragmented time, while real-time monitoring of training intensity through heart rate monitors or wearable devices enhances exercise efficiency. On rest days, moderate-intensity continuous aerobic training is recommended, with caution taken to avoid deterioration in technical form or excessive intensity due to accumulated fatigue. Post-exercise recovery strategies should be emphasised, and training plans adjusted promptly according to individual physical condition to ensure scientifically grounded and safe training.

### 4.2 Sleep characteristics of recreational runners

Sleep as a fundamental physiological need, plays an essential role in maintaining circadian rhythms, supporting physiological homeostasis, promoting psychological balance, and enhancing cognitive function (Phillips et al., 2017; Charest and Grandner, 2020). In this study, Recreational runners exhibited an average sleep onset time of 23:47 ± 1:17 and wake-up time of 6:34 ± 1:27, with a mean total sleep duration of 6.61 ± 0.69 h. Overall, sleep among recreational runners is insufficient, with average duration below the recommended 7 h. The National Sleep Foundation suggests 7–9 h for young adults (18–25 years) and 7–8 h for adults aged 26–64 years (Hirshkowitz et al., 2015). Limited time due to balancing training, work, and personal commitments may contribute to this insufficient sleep.

Analysis by gender revealed no significant differences in total sleep duration. However, female runners exhibited longer deep sleep duration, while male runners spent more time in awake duration, indicating lower sleep quality among males. This finding is consistent with previous research showing objectively superior sleep in females compared to males (Roberts et al., 2022). Deep sleep duration is a critical phase for bodily repair, memory consolidation, metabolic regulation, and physical recovery. It supports protein synthesis, cellular repair, and tissue regeneration, facilitating faster restoration of muscles, bones, and other tissues (Hobson, 2005). Prolonged awake duration reduces sleep quality. Therefore, male runners should prioritise maintaining sleep continuity to optimise recovery.

Sleep analysis across performance classifications revealed no significant differences in sleep architecture but indicated variations in sleep timing. Elite runners tended to have earlier sleep onset times and wake-up times, showing a pronounced early sleep-wake rhythm. This observation aligns with prior research. Given that marathon races often start between dawn and midday, circadian rhythm preferences may influence race-day performance. Previous studies using sleep chronotype questionnaires found that morning-type runners are overrepresented among marathon participants and generally perform better in races starting early, whereas later sleep preferences are associated with longer marathon completion times. Circadian rhythms are crucial in regulating physiological processes, behaviour, and performance (Anderson et al., 2018; Boivin et al., 2022; Nobari et al., 2023). Consequently, recreational runners should focus not only on total sleep duration and sleep quality but also on understanding and adapting their circadian rhythms. This includes adjusting schedules according to competition timing and implementing personalised rhythm-based training strategies to enhance race performance and physiological adaptability.

Seasonal sleep fluctuations among recreational runners were most pronounced during summer, when sleep quality tended to decline. Previous studies have reported total sleep duration to be approximately 30 min shorter in summer compared to autumn, likely due to longer daylight hours and higher ambient temperatures (Ferguson et al., 2021). Extended exposure to bright summer light suppresses melatonin secretion, delaying its release and shortening its duration, which disrupts circadian timing systems. Elevated temperatures further compromise sleep continuity and architecture by increasing thermal stress and reducing deep sleep duration, particularly REM duration, which is essential for cognitive function and physical recovery (Seidler et al., 2023).

Constrained by daily schedules and work commitments, many recreational runners have developed the habit of waking early. Data indicated that runners typically had wake-up times before 7:00 a.m. on both weekdays and rest days, with weekday wake-up times occurring earlier than on rest days. Previous research has shown that both the general population and professional athletes tend to use rest days to compensate for accumulated sleep deficits, resulting in longer total sleep duration on rest days (Owens and Adolescent Sleep Working Groupand Committee on Adolescence, 2014; Thun et al., 2015). However, findings from this study revealed that recreational runners slept longer on weekdays than on rest days. This discrepancy may reflect the recreational nature of recreational running, where training volume and intensity are lower on weekdays but are often increased on rest days without adequate recovery. Such practices can impair autonomic nervous system regulation and reduce sleep quality, as indicated by shorter deep sleep duration, shorter REM duration, and increased awake duration on rest days compared to weekdays. Therefore, recreational runners should be guided to plan training and recovery schedules rationally. Excessive training on rest days should be avoided, while maintaining regular sleep onset time, wake-up time, and sufficient total sleep duration to promote recovery and enhance long-term athletic performance.

### 4.3 Bidirectional relationships in recreational runners

Training intensity in this study was categorised as light training, moderate training, or high-volume training based on individual runner training mileage percentiles. A linear mixed model was employed to examine the effects of training on sleep quality during the subsequent night. Statistically significant effects of training on total sleep duration, deep sleep duration, light sleep duration, REM duration, and awake duration were observed. As training volume increased from light to high, device-estimated nocturnal HRV metrics showed a progressive decline, suggesting alterations in autonomic regulation and increased recovery-related demands. Alterations in sleep architecture were evident, with reductions in deep sleep duration and REM duration, alongside increases in light sleep duration and awake duration. Nevertheless, total sleep duration showed a marked increase, suggesting that recreational runners may partially compensate for reduced sleep quality by prolonging total sleep duration under higher training loads. It should be noted that although statistical significance was observed, effect sizes were small, indicating that the practical impact of training on recreational runner sleep patterns is limited. In contrast, previous studies on professional athletes have frequently reported that high-intensity or excessive training induces more pronounced sleep disturbances (Hausswirth et al., 2014; Dumortier et al., 2018). Hausswirth et al. (2014) divided 27 well-trained triathletes into overtraining and normal training groups, and 6-week continuous sleep monitoring revealed significantly reduced total sleep duration and sleep efficiency in the overtraining group. The relatively modest effects observed in recreational runners may reflect that typical training loads do not yet reach levels sufficient to exert a strong physiological impact on sleep. In contrast, elite athletes, due to extremely high training intensity and substantial recovery demands, exhibit greater sensitivity of sleep to training, resulting in more pronounced alterations in objective sleep metrics.

A bidirectional relationship may exist between training and sleep. On one hand, training load may affect total sleep duration and sleep architecture during the same night. On the other hand, insufficient sleep or reduced sleep quality may adversely affect subsequent training performance (Meeusen et al., 2021; Bestwick-Stevenson et al., 2022). Previous studies have reported that sleep deprivation in athletes negatively impacts physical performance while increasing the incidence and severity of musculoskeletal injuries (Yang et al., 2019; Nikolaidis et al., 2021; Karina et al., 2025; Veronica et al., 2025). Veronica et al. (2025) conducted the latest qualitative analysis on 31 Canadian competitive athletes with a history of injuries, indicating that athletes who experience difficulties falling asleep or maintaining sleep not only significantly increase their risk of injury but also suffer from emotional distress such as anxiety and shame. Meanwhile, Karina et al. (2025) carried out a quantitative study involving 385 Canadian athletes, which confirmed that poor sleep quality is significantly positively correlated with slower reaction times and serves as an important independent predictor of impaired cognitive-motor function in athletes. Together, these two studies, from both qualitative and quantitative perspectives, highlight the multifaceted potential harms of insufficient sleep for athletes’ maintenance of competitive status and prevention of sports injuries. In long-distance running specifically, Raghunandan et al. (2021) highlighted the importance of adequate sleep in preventing injuries among long-distance runners. Accordingly, recreational runners should be aware of the critical role of sleep. In this study, total sleep duration was classified into three categories: severe sleep deprivation (less than 6 h), mivld sleep deprivation (6–7 h), and normal sleep (over 7 h), in order to examine their effects on subsequent training performance. Sleep duration <6 h was defined as partial sleep deprivation, a state that has been proven to impair aerobic endurance, increase subsequent subjective fatigue, and reduce the training load voluntarily tolerated by athletes (Elmenhorst et al., 2008; Grandner et al., 2010; Dutil et al., 2025). The 7 h threshold is derived from the recommended guidelines of the American Academy of Sleep Medicine, which explicitly state that the recommended sleep duration is 7–9 h for individuals aged 18–25 years and 7–8 h for adults aged 26–64 years (Hirshkowitz et al., 2015), and this is consistent with the age distribution of recreational runners in the present study. Results indicated that sleep deprivation led to longer running duration, greater distance covered, and higher total step counts. However, at equivalent heart rate loads, pace was reduced and running efficiency decreased slightly. These findings suggest that sleep-deprived runners may compensate by prolonging training duration to maintain training volume, while completing the same physiological load at a slower pace. This reduction in training efficiency may be linked to central nervous system fatigue, delayed neuromuscular responses, or decreased energy metabolism. Moreover, increasing training volume under conditions of sleep deprivation does not necessarily improve training quality; instead, it may result in accumulated fatigue and inadequate recovery, thereby increasing the risk of injuries and reducing long-term training adaptations.

Although statistical analysis at the group level confirms a real association between training and sleep, the small effect size indicates limitations of group-average analysis. Individuals differ in training tolerance, recovery capacity, and lifestyle. These differences may mask meaningful individual responses during group analysis. In this context, the case study presented in the current research provides an important supplement. By using rolling historical data to establish baselines specific to individual runners, changes in training volume, sleep duration, and nocturnal HRV can be interpreted based on the inherent physiological norms of each individual rather than group reference values. This approach helps identify subtle abnormalities at an earlier stage when short-term training load exceeds the usual tolerance level of an individual. In addition, a specific pattern emerged from the case study. Training load increased continuously, while sleep and HRV decreased with a delay. This finding highlights the importance of dynamic, time-related interpretation for wearable device data. Personalized monitoring based on cumulative load and delayed physiological responses is more effective for identifying early signs of functional overreaching than responses to isolated daily fluctuations. Conversely, periods when training load decreases and sleep and HRV show corresponding recovery demonstrate that wearable device data can identify optimal recovery windows and potential supercompensation processes.

Taken together, these results show that there is an inseparable relationship between training, sleep and recovery. For recreational runners, improving performance only by adjusting training load is often difficult to achieve long-term and stable results. Only by focusing on training arrangements and paying attention to sleep quality and recovery status can we give full play to the adaptive effect of training stimulation and avoid fatigue accumulation and maladaptation. In this context, wearable technology provides a practical monitoring tool for recreational runners. By continuously recording training load, sleep behaviour and recovery-related indicators such as HRV, runners can gradually understand their own individual response characteristics to training stimulation. If these data are interpreted in combination with individual historical baselines, rather than simply compared with group average levels, it will be more helpful to adjust training and recovery strategies in a timely manner. Therefore, integrating wearable device-based monitoring into daily training practice is expected to become an important way for recreational runners to achieve personalized and sustainable progress.

### 4.4 Limitations

Although this study systematically analysed the training and sleep characteristics of Recreational runners using long-term, objective data from wearable devices, several limitations should be acknowledged. First, despite a substantial sample size, participants were limited to runners who voluntarily used Garmin devices and attended training camps. This may have introduced self-selection bias, reducing the generalisability of the findings to the wider population of recreational runners. Second, potential confounding factors, including occupational stress, shift work, family responsibilities, mental health status, dietary and caffeine intake, and electronic device usage—were not systematically collected or controlled. These factors may influence both training behaviour and sleep patterns, potentially distorting the true relationship between training and sleep. Future research should incorporate questionnaire-based assessments of lifestyle and psychological variables and adjust for these factors in statistical models to improve the precision of conclusions. Finally, although the longitudinal design partially captures temporal associations between training and sleep, it remains observational and cannot establish causal relationships. Future studies could employ randomised controlled trials to determine whether changes in training load affect sleep, or whether interventions aimed at improving sleep influence subsequent training performance, thereby clarifying causal mechanisms.

## 5 Conclusion

Based on 1-year longitudinal monitoring data, this study initially reveals the core characteristics of training and sleep among Chinese recreational runners, as well as their bidirectional temporal correlation, providing exploratory evidence for understanding the dynamic balance of training-recovery in recreational runners under real-world conditions. Significant variations in training were observed across gender, performance classification, season, and workday versus restday. Male runners exhibited higher training volume and faster pace than female runners. Training pace and volume increased with performance classification. Summer heat was associated with reduced training volume and slower pace, and training volume was lower on workdays compared to restdays. Sleep patterns revealed generally shorter total sleep duration among recreational runners, with differences in sleep architecture according to gender and performance classification. Female runners demonstrated objectively higher sleep quality than male runners, while elite-level runners exhibited a more pronounced “early to bed, early to rise” pattern. A statistically significant but modest bidirectional relationship was observed between training and sleep. High training volume was associated with fragmented sleep architecture and reduced autonomic nervous system recovery efficiency, whereas insufficient sleep was linked to decreased training efficiency and greater fatigue accumulation on subsequent days.

## Data Availability

The raw data supporting the conclusions of this article will be made available by the authors, without undue reservation.
