# Supplementary material for: Exploring training-sleep characteristics and bidirectional lagged relationships in Chinese recreational runners: insights from a year-long wearable monitoring study
Source: Front Physiol. 2026 Feb 20;17:1730135. doi: 10.3389/fphys.2026.1730135 (PMC12962949; doi:10.3389/fphys.2026.1730135)
Supplement: Supplementary file 1 [file Table1.docx]

**2025 Chinese Athletics Association marathon performance grading standards**

1. **Under 34 Years Old**

| Group/Grade/Event | Marathon | | Half Marathon | | 10km | | 5km | |
| --- | --- | --- | --- | --- | --- | --- | --- | --- |
|  | Male | Female | Male | Female | Male | Female | Male | Female |
| Elite Level | 3:00:00 | 3:20:00 | 1:25:00 | 1:35:00 | 0:37:00 | 0:47:00 | 0:18:00 | 0:21:00 |
| Level 1 | 3:30:00 | 3:50:00 | 1:35:00 | 1:45:00 | 0:42:00 | 0:52:00 | 0:21:00 | 0:24:00 |
| Level 2 | 4:00:00 | 4:20:00 | 1:55:00 | 2:05:00 | 0:52:00 | 1:02:00 | 0:26:00 | 0:29:00 |

1. **35-39 Years Old**

| Group/Grade/Event | Marathon | | Half Marathon | | 10km | | 5km | |
| --- | --- | --- | --- | --- | --- | --- | --- | --- |
|  | Male | Female | Male | Female | Male | Female | Male | Female |
| Elite Level | 3:10:00 | 3:30:00 | 1:30:00 | 1:40:00 | 0:38:00 | 0:48:00 | 0:19:00 | 0:22:00 |
| Level 1 | 3:40:00 | 4:00:00 | 1:40:00 | 1:50:00 | 0:43:00 | 0:53:00 | 0:22:00 | 0:25:00 |
| Level 2 | 4:10:00 | 4:30:00 | 2:00:00 | 2:10:00 | 0:53:00 | 1:03:00 | 0:27:00 | 0:30:00 |

1. **40-44 Years Old**

| Group/Grade/Event | Marathon | | Half Marathon | | 10km | | 5km | |
| --- | --- | --- | --- | --- | --- | --- | --- | --- |
|  | Male | Female | Male | Female | Male | Female | Male | Female |
| Elite Level | 3:15:00 | 3:35:00 | 1:32:00 | 1:42:00 | 0:39:00 | 0:49:00 | 0:20:00 | 0:23:00 |
| Level 1 | 3:45:00 | 4:05:00 | 1:42:00 | 1:52:00 | 0:44:00 | 0:54:00 | 0:23:00 | 0:26:00 |
| Level 2 | 4:15:00 | 4:35:00 | 2:02:00 | 2:12:00 | 0:54:00 | 1:04:00 | 0:28:00 | 0:31:00 |

1. **45-49 Years Old**

| Group/Grade/Event | Marathon | | Half Marathon | | 10km | | 5km | |
| --- | --- | --- | --- | --- | --- | --- | --- | --- |
|  | Male | Female | Male | Female | Male | Female | Male | Female |
| Elite Level | 3:20:00 | 3:40:00 | 1:35:00 | 1:45:00 | 0:40:00 | 0:50:00 | 0:21:00 | 0:24:00 |
| Level 1 | 3:50:00 | 4:10:00 | 1:45:00 | 1:55:00 | 0:45:00 | 0:55:00 | 0:24:00 | 0:27:00 |
| Level 2 | 4:20:00 | 4:40:00 | 2:05:00 | 2:15:00 | 0:55:00 | 1:05:00 | 0:29:00 | 0:32:00 |

1. **50-54 Years Old**

| Group/Grade/Event | Marathon | | Half Marathon | | 10km | | 5km | |
| --- | --- | --- | --- | --- | --- | --- | --- | --- |
|  | Male | Female | Male | Female | Male | Female | Male | Female |
| Elite Level | 3:30:00 | 3:50:00 | 1:40:00 | 1:50:00 | 0:41:00 | 0:51:00 | 0:22:00 | 0:25:00 |
| Level 1 | 4:00:00 | 4:20:00 | 1:50:00 | 2:00:00 | 0:46:00 | 0:56:00 | 0:25:00 | 0:28:00 |
| Level 2 | 4:30:00 | 4:50:00 | 2:10:00 | 2:20:00 | 0:56:00 | 1:06:00 | 0:30:00 | 0:33:00 |

1. **55-59 Years Old**

| Group/Grade/Event | Marathon | | Half Marathon | | 10km | | 5km | |
| --- | --- | --- | --- | --- | --- | --- | --- | --- |
|  | Male | Female | Male | Female | Male | Female | Male | Female |
| Elite Level | 3:40:00 | 4:00:00 | 1:45:00 | 1:55:00 | 0:42:00 | 0:52:00 | 0:23:00 | 0:26:00 |
| Level 1 | 4:10:00 | 4:30:00 | 1:55:00 | 2:05:00 | 0:47:00 | 0:57:00 | 0:26:00 | 0:29:00 |
| Level 2 | 4:40:00 | 5:00:00 | 2:15:00 | 2:25:00 | 0:57:00 | 1:07:00 | 0:31:00 | 0:34:00 |

1. **60-64 Years Old**

| Group/Grade/Event | Marathon | | Half Marathon | | 10km | | 5km | |
| --- | --- | --- | --- | --- | --- | --- | --- | --- |
|  | Male | Female | Male | Female | Male | Female | Male | Female |
| Elite Level | 3:50:00 | 4:20:00 | 1:50:00 | 2:05:00 | 0:43:00 | 0:53:00 | 0:24:00 | 0:27:00 |
| Level 1 | 4:20:00 | 4:50:00 | 2:00:00 | 2:15:00 | 0:48:00 | 0:58:00 | 0:27:00 | 0:30:00 |
| Level 2 | 4:50:00 | 5:20:00 | 2:20:00 | 2:35:00 | 0:58:00 | 1:08:00 | 0:32:00 | 0:35:00 |

**8. 65+ Years Old**

| Group/Grade/Event | Marathon | | Half Marathon | | 10km | | 5km | |
| --- | --- | --- | --- | --- | --- | --- | --- | --- |
|  | Male | Female | Male | Female | Male | Female | Male | Female |
| Elite Level | 4:00:00 | 4:30:00 | 1:55:00 | 2:10:00 | 0:44:00 | 0:54:00 | 0:25:00 | 0:28:00 |
| Level 1 | 4:30:00 | 5:00:00 | 2:05:00 | 2:20:00 | 0:49:00 | 0:59:00 | 0:28:00 | 0:31:00 |
| Level 2 | 5:00:00 | 5:30:00 | 2:25:00 | 2:40:00 | 0:59:00 | 1:09:00 | 0:33:00 | 0:36:00 |
